# An empirical analysis of overall survival in drug approvals by the US FDA (2006–2023)

**DOI:** 10.1002/cam4.7190

**Published:** 2024-04-25

**Authors:** Josh Elbaz, Alyson Haslam, Vinay Prasad

**Affiliations:** ^1^ Hofstra University Hempstead New York USA; ^2^ University of California San Francisco San Francisco California USA

**Keywords:** drug approval, FDA, oncology, overall survival

## Abstract

**Background:**

The US Food and Drug Administration (FDA) has expanded the use of surrogate markers in drugs approved for oncology/hematology indications. This has likely resulted in a greater number of approvals and possibly drugs coming to market faster, but it is unknown whether these drugs also improve overall survival (OS) for patients taking them.

**Methods:**

We sought to estimate the percentage of oncology drugs that have shown to improve OS in a cross‐sectional analysis of US FDA oncology drug approvals (2006–2023). We searched for OS data in registration trials and the peer‐reviewed literature.

**Results:**

We found 392 oncology drug approvals. Eighty‐seven (22%) drug approvals were based on OS, 147 drug approvals were later tested for OS benefit (38% of all approvals and 48% of drugs approved on a surrogate), and 130 (33%) have yet to be tested for OS benefit. Of the 147 drug approvals later tested for OS, 109 (28% of all approvals and 74% of drugs later tested for OS) have yet to show OS benefit, whereas 38 (10% of all approvals and 26% of drugs later tested for OS benefit) were later shown to have OS benefit. In total, 125 out of 392 (32%) drugs approved for any indication have been shown to improve OS benefit at some point, and 267 (68%) have yet to show approval.

**Conclusion:**

About 32% of all oncology drug approvals have evidence for an improvement in OS. Higher standards are needed in drug regulation to ensure that approved drugs are delivering better patient outcomes, specifically in regards to survival.

## 1 INTRODUCTION

Cancer drugs are approved in the United States (US) based on improvements in survival or quality of life, or surrogate outcomes that are expected to predict these clinical benefits. Response rate—the fraction of patients with 30% or more tumor shrinkage—and progression free survival—a composite time to event endpoint of death or tumor progression, are the most common surrogate endpoints used.^1^

Prior work has shown that the US Food and Drug Administration (FDA) has steadily expanded surrogate categories for drug approval over the last two decades.^2^ Moreover, surrogates are used for both accelerated approval, where there is a further post‐marketing efficacy commitment, and regular approval, where there is not.^3^ When used in this fashion, the FDA has authorized drugs based on surrogates with weak or unclear correlations with living longer or living better.

A prior analysis of 5 years of approvals (2008–2012) found that 67% were made on the basis of a surrogate endpoint and, after 4.5 years on the US market, most of the approvals (86%) either had unknown effects on overall survival (OS) or failed to show gains in survival.^4^ Since then, the number of approvals has increased, including the number of accelerated approvals,^5^ which require post‐marketing commitments to show clinical benefit. Concomitantly, the number and percentage of drugs approved on surrogate outcomes has also increased,^1^, ^6^ which limits the ability to draw conclusions on clinical benefit for patients, especially regarding OS.

The purpose of this study is to determine the frequency that cancer drugs are approved on OS or surrogate endpoints in the last two decades, and when approved on surrogate endpoints, to determine how many are tested for OS benefit and eventually demonstrate OS benefit.

## 2 METHODS

A retrospective review of cancer drugs approved from September 23, 2006 to January 10, 2023 was conducted using the FDA website and a prior systematic analysis of FDA approvals. We included all approvals for hematology and oncology malignancies for drugs used for an antitumor indication. Regular approvals for drugs that had initially received accelerated approval were considered duplicates and were removed. Data on approval characteristics (approval date and indication) as well as pivotal trial characteristics (demographics, primary endpoint, randomization, phase, blinding, and comparators) and results were abstracted from FDA announcement, drug labels, and Clinicaltrials.gov. Drugs that had received approval but were later withdrawn from the market, either from the FDA or voluntarily, were included in the analysis and were categorized as such.

We abstracted data on approval indication, approval outcome and supporting trial information (patient demographics, sample size, NCT number, trial name, primary and secondary endpoints and outcomes, phase, blinding, and randomization). Treatment setting and line (e.g., adjuvant, neoadjuvant, maintenance, and first and later lines) were abstracted from the FDA indication, as specified in the label or announcement. Drugs that were not indicated for adjuvant, neoadjuvant, or maintenance settings were coded as metastatic or not, and included advanced, unresectable, and relapse/refractory. Because of overlap in approval indications, we combined metastatic, advanced, unresectable, and relapsed/refractory into the broad category of “metastatic.”

When multiple trials supported approval, data were summarized as weighted percentages and average survival times (OS and PFS). Trial characteristics and demographic data were primarily abstracted from the respective FDA label. If trial data were missing from the FDA label (phase, blinding, and randomization), data were supplemented by the clinicaltrials.gov registration page. If median age and percentage of male/female were not recorded in the FDA label, we left these data blank, as abstraction from other sources, either publications or clinicaltrials.gov, may not be reflective of the state of the trial at the time of approval. Data were abstracted by two reviewers (JE and AH).

For approvals based on surrogate endpoints, a systematic search was conducted to identify and document OS data published after approval. PubMed was initially searched using the following terms: (1) [Drug name] AND [trial name] AND/OR [NCT number] AND “overall survival.” If nothing was found, the pivotal trial registration page was identified on clinicaltrials.gov and linked references were searched. If nothing was found again, a Google Search was conducted with the same terms used on PubMed. OS data from the registration trial (or confirmatory trial if approved via the accelerated pathway) used for approval was prioritized, but if OS data were unavailable, the search was expanded to include any other studies testing the drug for the same indication listed in the FDA announcement/label (could be phase II or phase III trials). To give the most opportunity for a drug to show OS benefit, they were considered to show OS benefit if any non‐registration trial was positive, even if others did not. Overall survival data were only recorded from peer‐reviewed publications; abstracts, posters, webpages, and presentations were not included. From these trials, we abstracted data on OS (median for each arm, hazard ratio, and statistical significance) and date of first publication.

When assessing trials with comparator arms, the terms standard of care, basic supportive care, and supportive care were all considered “placebo.” Drugs approved on surrogate endpoints were considered tested for OS benefit if we found a publication reporting specific OS data from a randomized trial for the same indication as the approval. Thus, drugs tested in single‐arm trials received no credit for testing or improving OS. Statistical significance of OS results was based on the HR, the a priori statistical significance level for the trial, and whether the OS data were mature. We assumed that OS data were mature, unless the trial report indicated otherwise. Tested with OS benefit was counted when all of the following criteria were met: (1) drug approved on surrogate endpoint, (2) ever tested for OS benefit, (3) OS results were statistically significant, and (4) OS results were mature.

### 2.1 Statistical methods

Data were summarized as descriptive characteristics, with frequencies and percentages for categorical variables and medians and interquartile ranges (IQR) for continuous variables. We used R statistical software (version 4.2.0) for all statistical analysis and figure creation.

In accordance with 45 CFR §46.102(f), this study was not submitted for University of California, San Francisco institutional review board approval because it involved publicly available data and did not involve individual patient data.

## 3 RESULTS

We identified 392 unique drug authorizations, 28 (7%) of which were eventually withdrawn from the market. Table 1 shows the characteristics of these approvals. Among included drugs, 335 (85%) were for metastatic indications, 24 (6%) were for nonmetastatic indications, 17 (5%) were for adjuvant indications, 13 (3%) were for maintenance indications, and 3 (1%) were for neoadjuvant indications. No single‐arm trials were done to support approval in the adjuvant, neoadjuvant, and maintenance settings. In total, 115 (29%) were initially approved via the accelerated pathway, the majority of accelerated approvals were granted on single‐arm data.

**TABLE 1:** Approval characteristics of drug approvals by the US Food and Drug Administration (2006 through January 2023).

Approval characteristics	*N*	All approvals	Single arm	Randomized	*p*‐Value^b^
		*N* = 392^a^	*N* = 149^a^	*N* = 243^a^	
Withdrawn	392				<0.001
No		364 (93%)	126 (85%)	238 (98%)	
Yes		28 (7%)	22 (15%)	6 (2%)	
Treatment setting	392				<0.001
Metastatic		335 (85%)	136 (91%)	199 (82%)	
Nonmetastatic		24 (6%)	13 (8%)	11 (5%)	
Adjuvant		17 (4%)	–	17 (7%)	
Maintenance		13 (3%)	–	13 (5%)	
Neoadjuvant		3 (1%)	–	3 (11%)	
Accelerated approval	392				<0.001
No		277 (71%)	58 (39%)	219 (90%)	
Yes		115 (29%)	91 (61%)	24 (10%)	
Tumor type	392				<0.001
Hematologic		120 (31%)	71 (48%)	49 (20%)	
Reproductive		67 (17%)	13 (9%)	54 (22%)	
Lung		61 (16%)	20 (8%)	41 (17%)	
GI		45 (11%)	9 (6%)	36 (8%)	
Urinary tract		29 (7.4%)	10 (7%)	19 (8%)	
Skin		24 (6.1%)	4 (3%)	20 (8%)	
Other		16 (4.1%)	6 (40%)	10 (41%)	
Tumor agnostic		15 (3.8%)	11 (7%)	4 (2%)	
Head and neck		11 (2.8%)	3 (2%)	8 (3%)	
Nervous		4 (1.0%)	2 (1%)	2 (1%)	
Phase	392				<0.001
1		18 (4.6%)	18 (12%)	–	
1/2		39 (9.9%)	35 (23%)	4 (10%)	
2		90 (23%)	75 (50%)	15 (17%)	
2/3		6 (2%)	4 (3%)	2 (1%)	
3		226 (58%)	5 (3%)	221 (91%)	
Unknown		13 (3%)	12 (8%)	1 (<1%)	
Randomized	392				<0.001
Non‐randomized		149 (38%)	149 (100%)	0 (0%)	
Randomized		243 (62%)	–	243 (100%)	
Randomization ratio	243				
1:1		173 (71%)	–	173 (71%)	
2:1		67 (28%)	–	67 (28%)	
3:1		1 (0.4%)	–	1 (<1%)	
3:2		2 (0.8%)	–	2 (1%)	
Blinding	392				<0.001
None (Open label)		283 (72%)	149 (100%)	134 (56%)	
Single		4 (1.0%)	–	4 (2%)	
Double		61 (16%)	–	61 (25%)	
Triple		13 (3%)	–	13 (3%)	
Quadruple		31 (8%)	–	31 (13%)	
Comparator	248				0.2
Non‐placebo		110 (45%)	5 (3%)	105 (43%)	
Placebo + other therapy		58 (23%)	–	58 (24%)	
Placebo only		80 (32%)	–	80 (33%)	
Approved on OS	392				<0.001
No		304 (78%)	149 (100%)	155 (64%)	
Yes		88 (22%)	–	88 (36%)	
Primary endpoint	392				<0.001
ORR		134 (34%)	119 (78%)	15 (6%)	
PFS		101 (26%)	–	101 (42%)	
OS		88 (22%)	–	88 (36%)	
CR		26 (7%)	18 (12%)	8 (3%)	
DFS/MFS/EFS		20 (5%)	–	20 (8%)	
Other		15 (4%)	10 (27%)	5 (2%)	
RFS		7 (2%)	–	7 (3%)	
MRD		1 (<1%)	1 (1%)	‐	

*Note*: **Tumor types key**: Skin: basal cell carcinoma, melanoma, Merkel. Gastrointestinal: esophageal, biliary, cholangiocarcinoma, CRC, gastroesophageal, HCC, pancreatic. Lung: mesothelioma, mesothelioma, NSCLC, SCLC. Head and neck; HNSCC, thyroid. Hematologic: AML, follicular lymphoma, Hodgkin lymphoma, inflammatory anaplastic lymphoma, MDS, large B‐cell lymphoma (LBCL), leukemia, leukemia/lymphoma, lymphoma, multiple myeloma, myeloid/lymphoid neoplasms, myelodysplastic syndrome, chronic lymphocytic leukemia. Nervous: glioblastoma, dendritic cell neoplasm, neuroblastoma. Reproductive: cervical, cervical, breast, endometrial, ovarian, prostate. Urinary tract: bladder, RCC, urothelial. Other: amyloidosis, epithelioid cell tumor, giant cell tumor of bone, GIST, Kaposi sarcoma, neuroendocrine, tenosynovial giant cell tumor, Waldenstrom's macroglobulinemia, Waldenstrom's macroglobulinemia. Tumor agnostic: MMR solid tumors, MSI solid tumors, NTRK solid tumors, sarcoma, solid tumors, squamous cell carcinoma, TMB solid tumors.

^*a*^ *n* (%); median (IQR).

^*b*^ Wilcoxon rank sum test; Pearson's chi‐squared test.

The most common tumor types were broadly included: 120 (31%) hematologic, 67 (17%) reproductive, and 61 (16%) lung tumors. Hematologic and tumor agnostic approvals were the only groups where a majority of approvals was granted on the basis of single‐arm data—59% and 73%, respectively.

The majority of trials were phase 3 (*n* = 226; 58%), randomized (*n* = 243; 62%), and not blinded (*n* = 277; 72%). Among the 243 approvals based on randomized studies, 173 (71%) used a 1:1 ratio. For trials with comparator arms, 105 (43%) used comparators which were non‐placebo, 80 (33%) placebo alone, and 58 (24%) used placebo in combination with another therapy.

Table 2 describes the characteristics of the studies used for the approvals. Median age for trial participants was 62 (IQR: 57, 65), and median percentage of enrolled males was 58% (IQR: 46, 71) compared to 42% (IQR: 29, 54) for females. Median number of individuals randomized to intervention versus control arms were 191 (IQR: 101, 329) and 229 (IQR: 134, 354), respectively. Randomized trials had a significantly higher number of participants in the intervention arm, compared to single‐arm trials: the median number of individuals was 282 (IQR: 181, 408) and 101 (IQR: 66, 138), respectively. Age and gender distributions were similar between randomized and single‐arm trials.

**TABLE 2:** Study characteristics of drug approvals by the US Food and Drug Administration (2006 through January 2023).

Trial results	*N*	All approvals	Single arm	Randomized	*p*‐Value^b^
		*N* = 392^a^	*N* = 149^a^	*N* = 243^a^	
Age	374	62 (57, 65)	62 (55, 66)	62 (58, 64)	0.9
Male	382	58 (46, 71)	57 (46, 69)	59 (45, 73)	0.5
Female	382	42 (29, 54)	43 (31, 54)	41 (27, 56)	0.5
Intervention arm	392	191 (101, 329)	101 (66, 138)	282 (181, 408)	<0.001
Control arm	241	229 (134, 354)	49 (49, 49)	231 (136, 355)	0.10
Original approval median OS—intervention arm	74	13.5 (10.3, 17.3)	–	13.5 (10.3, 17.3)	
Original approval median OS—control arm	85	11 (8, 14)	–	11 (8, 14)	
Original approval median HR	88	0.71 (0.66, 0.78)	–	0.71 (0.66, 0.78)	
Post approval median OS—intervention arm	135	19 (12, 29)	15 (9, 21)	24 (17, 39)	<0.001
Post approval median OS—control arm	105	19 (13, 32)	12 (9, 16)	20 (15, 36)	<0.001
Post approval median HR	153	0.78 (0.67, 0.89)	0.75 (0.55, 0.89)	0.80 (0.69, 0.89)	0.4
OS significance	239				0.2
No		108 (45%)	13 (9%)	95 (39%)	
Yes		131 (55%)	8 (5%)	123 (61%)	
Approval assessment	392				<0.001
Not tested		130 (33%)	108 (72%)	22 (9%)	
Ever tested + No OS benefit		109 (28%)	12 (8%)	97 (40%)	
Approved on OS (excluding withdrawn)		87 (22%)	–	87 (36%)	
Ever tested + OS benefit		38 (10%)	6 (4%)	32 (13%)	
Withdrawn		28 (7%)	22 (9%)	6 (2%)	

^*a*^ Median (IQR); *n* (%).

^*b*^ Wilcoxon rank sum test; Pearson's chi‐squared test.

Eighty‐seven (22%) were approved on OS (excluding one that was initially approved on OS and then withdrawn) and 305 (78%) on a surrogate endpoint. ORR was used in 34% of approvals (134 out of 392), and PFS was used in 26% (101 out of 392) approvals. Other endpoints used for approval were complete response (*n* = 26; 7%) event‐free/disease‐free/metastasis‐free survival (*n* = 20; 5%), and relapse‐free survival (*n* = 7; 2%). Among those approved on a surrogate endpoint, 115 (29%) were accelerated approvals.

Of the 87 drugs approved on OS and not withdrawn, the median overall survival for intervention and control arms were 13.4 months (IQR: 10.3, 17.1) and 11 months (IQR: 8, 14), respectively, with a median HR of 0.71 (IQR: 0.67, 0.78). Among those approved on a surrogate endpoint (*n* = 305), 153 (50%) were eventually tested for OS, 29 (8%) of which showed OS benefit. Most surrogate approvals that were later tested and did not show OS benefit were originally made on the basis of randomized trial data (*n* = 97; 89%), whereas those that were not eventually tested for OS benefit were mostly approved on single‐arm data (*n* = 108; 83%). Post approval median OS for intervention and control arms were 19 (13, 29) and 19 (13, 33), respectively, with a median HR of 0.78 (0.67, 0.89).

Of the 87 drugs originally approved on OS and that were not withdrawn, 82 (94%) were for the metastatic setting. Likewise, 106 (82%) of the single‐arm trials, 86 (79%) of surrogate approvals later tested for OS but no OS benefit, and 33 (87%) of those tested and subsequently showing OS benefit were approved for metastatic treatment. Table 3 shows the number of drugs approved on OS, by treatment setting.

**TABLE 3:** Frequency of drugs approved by overall survival testing and treatment setting.

	Single‐arm trials	Surrogate approval showing no OS benefit	Surrogate approval showing OS benefit	Drugs approved on OS	Withdrawn
	*N* = 130^a^	*N* = 109^a^	*N* = 38^a^	*N* = 87^a^	*N* = 28^a^
Treatment setting					
Metastatic	106 (82%)	86 (79%)	33 (87%)	82 (94%)	28 (100%)
Nonmetastatic	12 (9%)	7 (6%)	2 (5%)	2 (2%)	
Adjuvant	6 (5%)	10 (9%)	1 (3%)	0 (0%)	
Maintenance	4 (3%)	4 (4%)	2 (5%)	3 (3%)	
Neoadjuvant	1 (1%)	2 (2%)	0 (0%)	0 (0%)	

^*a*^ *n* (%).

In total, 87 (22%) drug approvals were based on OS, 147 drug approvals were later tested for OS benefit (38% of all approvals and 48% of drugs approved on a surrogate), and 155 (40%) were not tested for OS benefit. Of the 147 drug approvals later tested for OS, 109 (28% of all approvals and 74% of drugs later tested for OS) have yet to show OS benefit, whereas 38 (10% of all approvals and 26% of drugs later tested for OS benefit) were later shown to have OS benefit.

**FIGURE 1:**
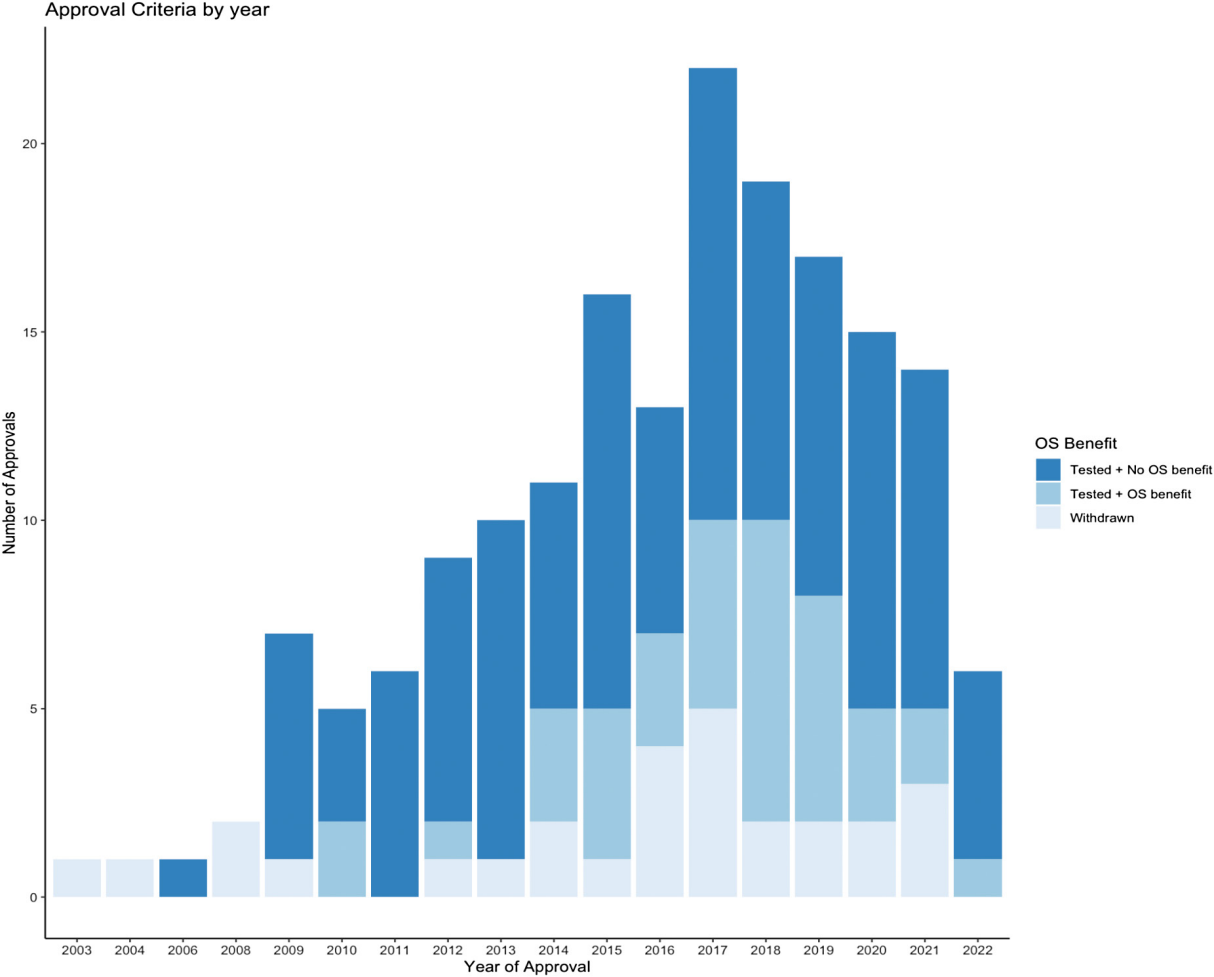
Drug approvals by the US Food and Drug Administration (2006 through January 2023), by year and overall survival (OS) testing status.

**FIGURE 2:**
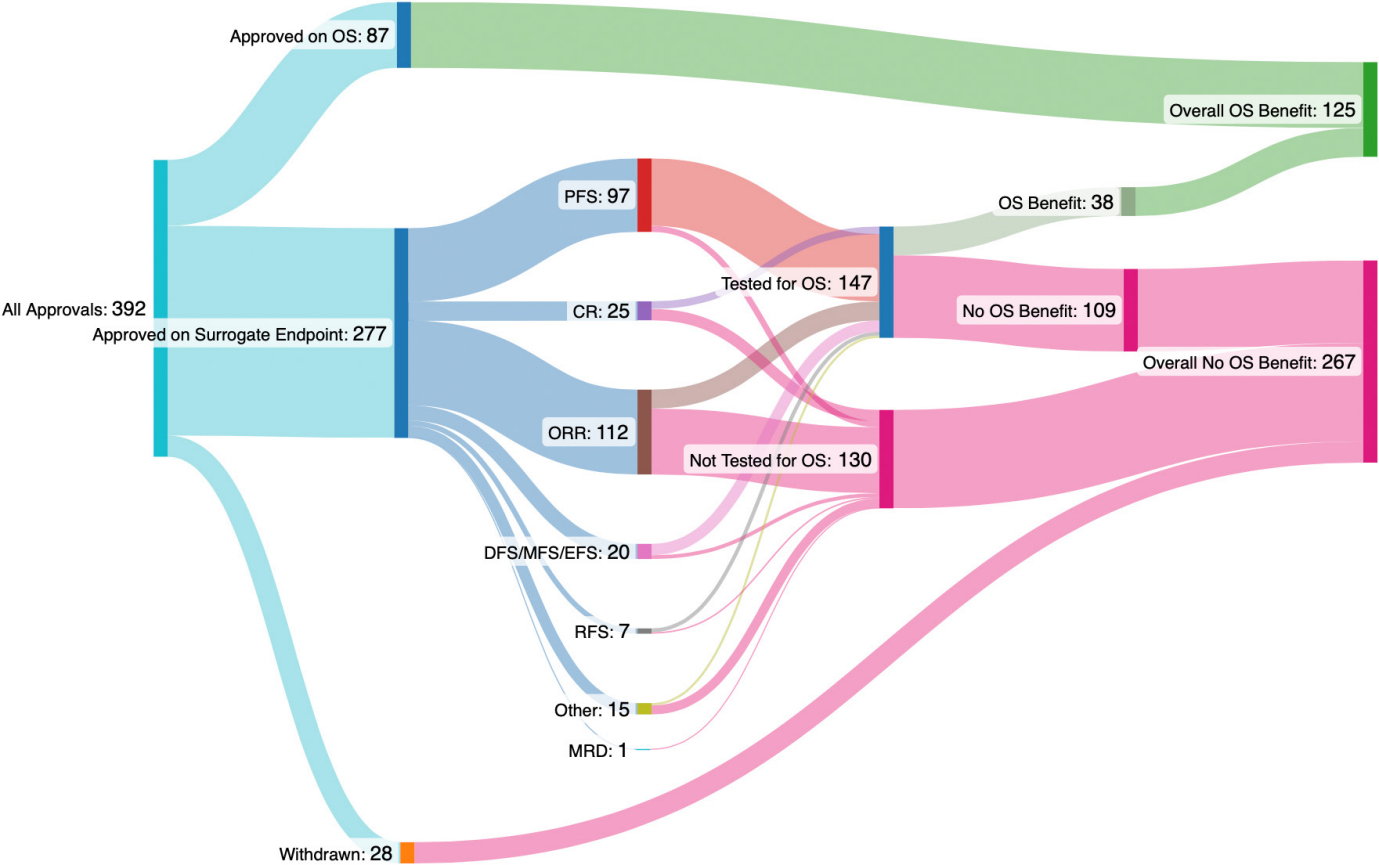
Flow diagram of oncology drug approvals, testing of overall survival, and demonstrating overall survival.

In total 125 out of 392 (32%) of drugs approved for any indication have been shown to improve OS benefit at some point, and 267 (68%) have yet to show survival benefit. Figures 1 and 2 show the numbers, by year of drugs approved, based on whether they improved OS or not. Figures S1 and S2 show OS status by year and treatment setting.

## 4 DISCUSSION

We found that among 392 oncology indication approvals, 32% had an OS benefit (either upon approval or in later studies), 28% of indication approvals were tested, but failed to show OS benefit, and 33% of indication approvals have yet to be tested for OS benefit, and 7% were withdrawn from the market.^7^, ^8^, ^9^, ^10^

The role of the FDA is to oversee and protect the health of people in the US, ensuring that the drugs Americans have access to are safe and effective. Clinical effectiveness in oncology refers only to drugs that improve survival or quality of life or both. Prior research has shown that few drugs improve quality of life in the absence of OS benefits.^11^, ^12^, ^13^ In other words, most drugs that improve quality of life also extend survival. For our analysis, we considered this, the gold standard endpoint, and found that the majority of FDA approvals fall short.

Although US drug regulation concerns matters specific to the US, it has implications globally. The US can set a precedent for available therapies in other countries, thus influencing care that is provided outside of the US. This is especially true in low‐to‐middle‐income countries who may lack the resources to do their own drug evaluation.^14^ The regulatory approval process should be designed to document patient benefit—if not at approval, at some point in the lifecycle—and if drugs fail to achieve this benchmark, arguably they should be removed from the market.

Most new drugs are approved in the US before they are approved by regulatory agencies of other countries.^15^, ^16^ Some might argue that this faster approval time allows patients earlier access to drugs and theoretically earlier access to benefit, but more drugs being approved on surrogates that are inadequate markers of patient benefit does not guarantee clinical benefit. Further, prior analyses have shown a lack of correlation between earlier approvals and clinical benefit, according to the European Society for Medical Oncology Magnitude of Clinical Benefit Scale.^15^

Recently, the FDA has issued guidance on strengthening accelerated approval. However, our papers document persistent limitations to this guidance. Specifically, PFS can be accepted as a confirmatory endpoint for drugs approved based on response rate, but PFS is itself not a direct measure of how well patients feel or function, often correlating poorly with these outcomes.^17^, ^18^ The effects on overall survival remain unknown for a majority of drugs. Our analysis suggests that more confirmatory trials should be designed and powered to assess survival.

The results support the notion that trials approved on the basis of single‐arm trials serve a unique role in the FDA drug approval process. Compared to approvals based on randomized data, these were disproportionately based on accelerated approvals and for hematologic cancers in the metastatic setting. Although they were also withdrawn from the market at a greater rate, this was not significant. Unsurprisingly, single‐arm approvals also depended more on earlier phase trials, the vast majority of which were phases 1–2.

The age and gender distributions of these approvals did not differ significantly, and as expected, single‐arm trials were significantly smaller. Although median OS increased in both control and intervention arms when tested post‐approval, an increase in median hazard ratio points to a diminished magnitude in OS benefit. Nevertheless, the vast majority (74%) of drugs approved on a surrogate endpoint and later tested for OS benefit yielded insignificant results. This inevitably raises the question of whether the standard of evidence for FDA drug approval is appropriate.

Compared to other studies, we similarly found that the percentage of drug approvals based on OS was small,^19^ and almost one‐third of approvals are via the accelerated pathway that requires post‐marketing studies.^20^ However, our study extends these findings by evaluating the fate of these approvals to see how many provide further evidence of OS efficacy, even among drugs receiving accelerated approval. Our findings suggest that even with confirmatory studies for accelerated approvals, most drugs have not been shown to improve OS.

### 4.1 Strengths and limitations

Our study is, to our knowledge, the most comprehensive analysis examining the fate of drugs approved for oncology indications, including approvals from 2006 to 2023. Moreover, we present results, stratified by time and treatment setting. A limitation in our study is that not all drug approvals may be captured. Our list of approvals was taken from the FDA oncology drug announcement website,^21^ Hemeonc.org, and prior studies of this nature. Combining these sources has allowed us to compile a comprehensive list of approvals. A second limitation is that we may not have captured all studies done to evaluate OS. We used several methods, including searching by trial name and by drug name and indication.

## 5 CONCLUSION

In conclusion, we found that less than one‐third of drug indication approvals by the FDA have been shown to improve OS with a median 5 years on the US market. Of the drugs that are approved on a surrogate, only about half are tested for survival benefit, yet only about 20% of the drugs not approved on a surrogate show OS benefit. Our results suggest persistent deficiencies in the US regulation of cancer drug products. Regulators are tolerating a high degree of uncertainty for these products.

## AUTHOR CONTRIBUTIONS

**Josh Elbaz:** Conceptualization (equal); data curation (equal); formal analysis (equal); methodology (equal); writing – original draft (equal). **Alyson Haslam:** Conceptualization (equal); data curation (equal); methodology (equal); writing – review and editing (equal). **Vinay Prasad:** Conceptualization (equal); supervision (equal); writing – review and editing (equal).

## FUNDING INFORMATION

Arnold Ventures.

## CONFLICT OF INTEREST STATEMENT

V.P. received research funding from Arnold Ventures through a grant made to UCSF, and royalties for books and writing from Johns Hopkins Press, MedPage, and the Free Press. He declared consultancy roles with UnitedHealthcare and OptumRX; He hosts the podcasts, Plenary Session, VPZD, Sensible Medicine, writes the newsletters, Sensible Medicine, the Drug Development Letter, and VP's Observations and Thoughts, and runs the YouTube channel Vinay Prasad MD MPH, which collectively earn revenue on the platforms: Patreon, YouTube, and Substack. JE and AH have no disclosures to report.

## Supporting information


**Figure S1.** Drug approvals by the US Food and Drug Administration (2006 through January 2023), by year and overall survival (OS) status.
**FIGURE S2.** Drug approvals by the US Food and Drug Administration (2006 through January 2023), by treatment setting and overall survival (OS) status.

## Data Availability

Data are available upon reasonable request to the author.
