# Supplementary material for: An empirical analysis of overall survival in drug approvals by the US FDA (2006–2023)
Source: Cancer Med. 2024 Apr 25;13(8):e7190. doi: 10.1002/cam4.7190 (PMC11043668; doi:10.1002/cam4.7190)
Supplement: Supplementary file 1 — Figure S1. Drug approvals by the US Food and Drug Administration (2006 through January 2023), by year and overall survival (OS) status. FIGURE S2. Drug approvals by the US Food and Drug Administration (2006 through January 2023), by treatment setting and overall survival (OS) status. [file CAM4-13-e7190-s001.docx]

**Supplemental Figure 1.** Drug approvals by the US Food and Drug Administration (2006 through January 2023, by year and overall survival (OS) status.

**
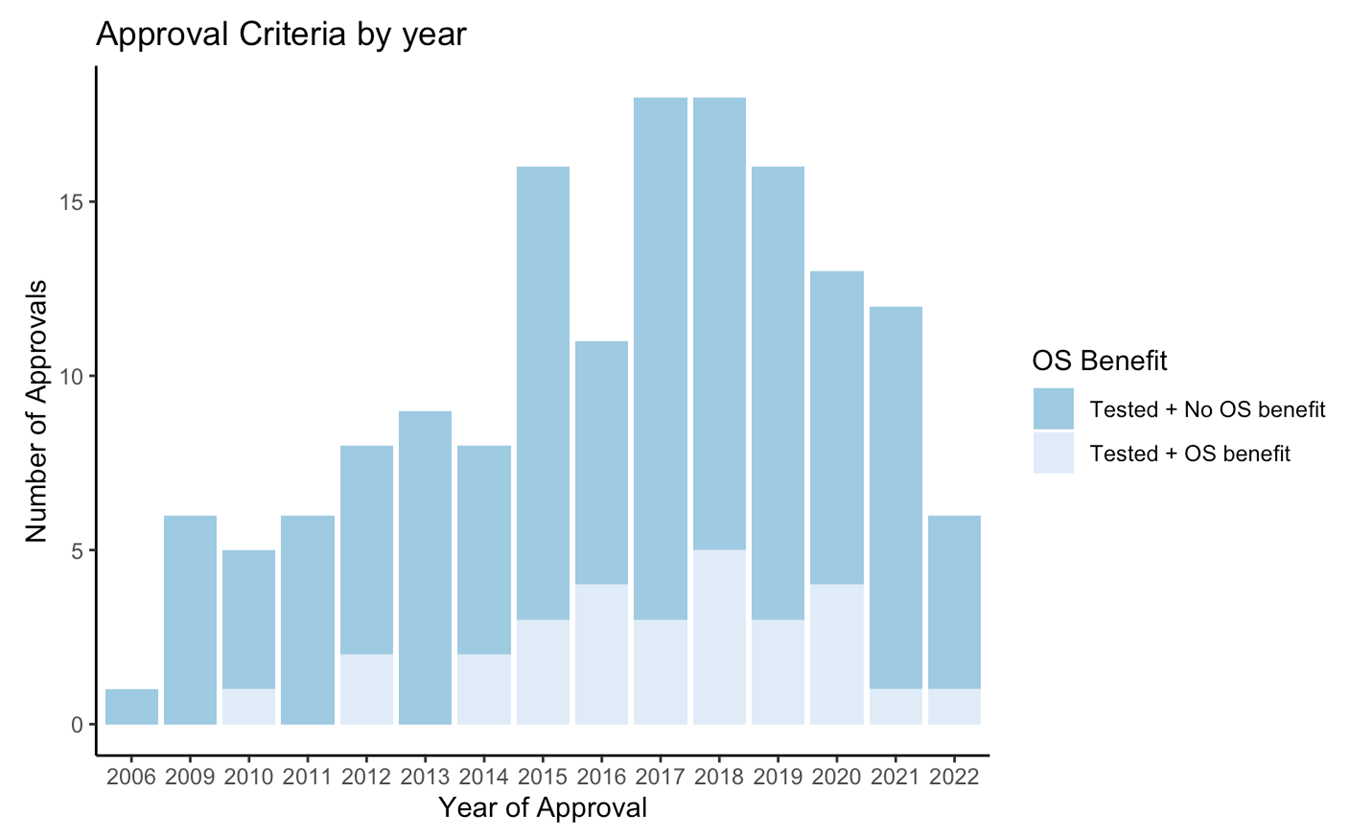
**

**Supplemental Figure 2.**Drug approvals by the US Food and Drug Administration (2006 through January 2023, by treatment setting and overall survival (OS) status.

**
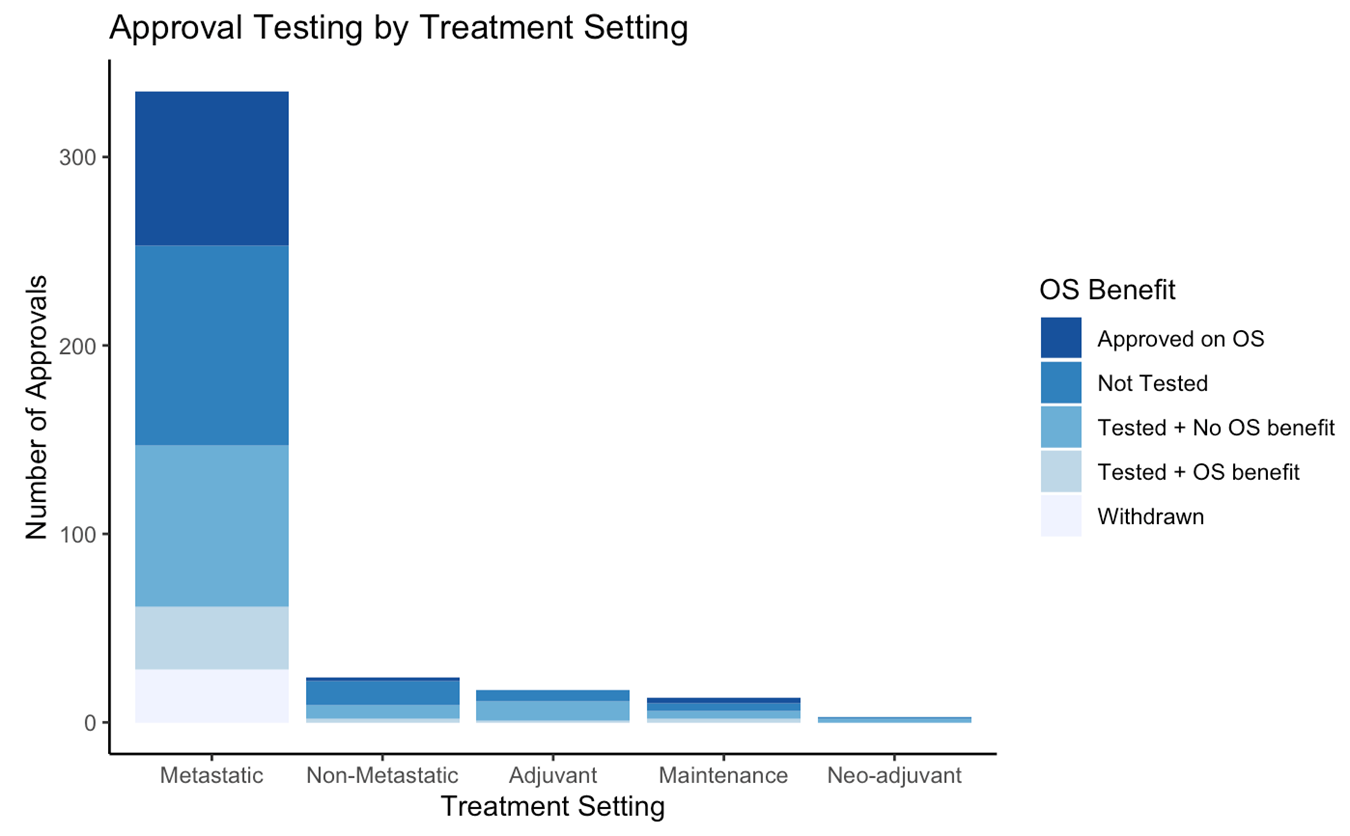
**
